# A Novel Bifunctional Amino Acid Racemase With Multiple Substrate Specificity, MalY From *Lactobacillus sakei* LT-13: Genome-Based Identification and Enzymological Characterization

**DOI:** 10.3389/fmicb.2018.00403

**Published:** 2018-03-07

**Authors:** Shiro Kato, Tadao Oikawa

**Affiliations:** ^1^High Technology Research Core, Kansai University, Suita, Japan; ^2^Department of Life Science and Biotechnology, Faculty of Chemistry, Materials, and Bioengineering, Kansai University, Suita, Japan

**Keywords:** D-amino acid, amino acid racemase, *Lactobacillus sakei*, genome analysis, lactic acid bacteria, maltose regulon, cystathionine β-lyase, bifunctional enzyme

## Abstract

The *Lactobacillus sakei* strain LK-145 isolated from Moto, a starter of sake, produces potentially large amounts of three D-amino acids, D-Ala, D-Glu, and D-Asp, in a medium containing amylase-digested rice as a carbon source. The comparison of metabolic pathways deduced from the complete genome sequence of strain LK-145 to the type culture strain of *Lactobacillus sakei* strain LT-13 showed that the L- and D-amino acid metabolic pathways are similar between the two strains. However, a marked difference was observed in the putative cysteine/methionine metabolic pathways of strain LK-145 and LT-13. The cystathionine β-lyase homolog gene *malY* was annotated only in the genome of strain LT-13. Cystathionine β-lyase is an important enzyme in the cysteine/methionine metabolic pathway that catalyzes the conversion of L-cystathionine into L-homocysteine. In addition to *malY*, most genome-sequenced strains of *L. sakei* including LT-13 lacked the homologous genes encoding other putative enzymes in this pathway. Accordingly, the cysteine/methionine metabolic pathway likely does not function well in almost all strains of *L. sakei*. We succeeded in cloning and expressing the *malY* gene from strain LT-13 (*Ls-malY*) in the cells of *Escherichia coli* BL21 (DE3) and characterized the enzymological properties of *Ls*-MalY. Spectral analysis of purified *Ls*-MalY showed that *Ls*-MalY contained a pyridoxal 5′-phosphate (PLP) as a cofactor, and this observation agreed well with the prediction based on its primary structure. *Ls*-MalY showed amino acid racemase activity and cystathionine β-lyase activity. *Ls*-MalY showed amino acid racemase activities in various amino acids, such as Ala, Arg, Asn, Glu, Gln, His, Leu, Lys, Met, Ser, Thr, Trp, and Val. Mutational analysis revealed that the 𝜀-amino group of Lys233 in the primary structure of *Ls*-MalY likely bound to PLP, and Lys233 was an essential residue for *Ls*-MalY to catalyze both the amino acid racemase and β-lyase reactions. In addition, Tyr123 was a catalytic residue in the amino acid racemase reaction but strongly affected β-lyase activity. These results showed that *Ls*-MalY is a novel bifunctional amino acid racemase with multiple substrate specificity; both the amino acid racemase and β-lyase reactions of *Ls*-MalY were catalyzed at the same active site.

## Introduction

A main source of D-amino acids in bacteria is amino acid racemase, which catalyzes the interconversion of D- and L-enantiomers of amino acids. In general, amino acid racemase is classified into two groups: pyridoxal 5′-phosphate (PLP)-independent enzyme and PLP-dependent enzyme. The PLP-independent amino acid racemase includes glutamate racemase (Choi et al., 1992; Yoshimura et al., 1993), aspartate racemase (Fujii et al., 2015), and proline racemase (Cardinale and Abeles, 1968) and contains two Cys residues as a catalytic residue (Choi et al., 1992; Washio et al., 2016). In contrast, PLP-dependent amino acid racemases such as alanine racemase (Oikawa et al., 2006) and arginine racemase (Matsui et al., 2009) requires PLP as a cofactor. Although amino acid racemases from various bacteria have been studied extensively, novel amino acid racemases may still be discovered. Li and Lu demonstrated that D-Arg was metabolized after racemization into L-Arg using a novel amino acid racemase consisting of two PLP-independent dehydrogenases in *Pseudomonas aeruginosa* (Li and Lu, 2009). A protein annotated as a γ-aminobutyrate aminotransferase from *Lactobacillus otakiensis* or *Lactobacillus buchneri* has been identified as a novel PLP-dependent epimerase that converts L-Ile into D-allo-Ile (Mutaguchi et al., 2013b). Recently, the RacX from *Bacillus subtilis* and YgeA from *Escherichia coli* MG1655 have been shown to be a novel amino acid racemase with broad substrate specificity (Miyamoto et al., 2017). The physiological roles of D-amino acid in bacteria have long been considered, but only as providing an essential component in bacterial peptidoglycan and antibiotics. However, recent studies of bacterial D-amino acids have revealed that some D-amino acids relate to other physiological roles, such as the remodeling of bacterial peptidoglycan in the stationary phase (Lam et al., 2009) and the dispersion of bacterial biofilm (Kolodkin-Gal et al., 2010). These attractive studies in the research field of D-amino acids motivate researchers such as ourselves to find a novel amino acid racemase and a novel role for D-amino acid in bacteria.

Lactic acid bacteria are Gram-positive lactic acid-producing bacteria and are used as starters in fermented foods such as Japanese sake, wine, vinegar, yogurt, and cheese. Several research groups, including our lab group, have clarified that fermented foods contain significant amount of D-amino acids, and such D-amino acids are mainly produced by lactic acid bacteria (Gogami et al., 2011; Kato et al., 2011; Mutaguchi et al., 2013a). Our group reported for the first time that D-Ala, D-Asp, and D-Glu in Japanese sake increase the taste and total balance of the taste of sake, and other D-amino acids showed no effect (Okada et al., 2013). Recently, we analyzed and reported the complete genome sequences of two *Lactobacillus sakei* strains, LK-145 (Kato and Oikawa, 2017a) and LT-13 (Kato and Oikawa, 2017b). Strain LK-145 was isolated from a Japanese sake seller as a high D-amino acid producer (Gogami et al., 2011) and strain LT-13 was isolated from Moto, a starter of sake, as a low D-amino acid producer, using a medium of amylase digested rice as a carbon source. The overall genome structure of strain LK-145 was similar to that of strain LT-13 (Kato and Oikawa, 2017a,b) or *L. sakei* strain 23K (Chaillou et al., 2005), the first genome sequenced strain of *L. sakei*. However, a marked difference was observed in the putative cysteine/methionine metabolic pathways of strain LK-145 and LT-13. The cystathionine β-lyase homolog gene (accession no. BAX66038), *malY* was only annotated in the genome of strain LT-13. Accordingly, the gene product of *malY* is expected to be involved in the differences in D-amino acid productivity between strain LK-145 and strain LT-13.

In this study, we tried to clone and express the *malY* gene from the genome of strain LT-13 (*Ls-malY*) in the cells of *E. coli* BL21 (DE3) and to characterize the enzyme properties of *Ls*-MalY *in vitro* to elucidate the relationship between *Ls*-MalY and the D-amino acid metabolism of strain LT-13.

## Materials and Methods

### Reagents

Amino acids and pyruvic acid were purchased from Wako Pure Chemicals, Co., Ltd. (Japan), Watanabe Chemical Industries, Ltd. (Japan) or Sigma Japan. Restriction enzymes were from New England Biolabs Japan. KOD -plus ver. 2 DNA polymerase was from Toyobo, Co., Ltd. (Japan). Methanol and acetonitrile were from Kanto Kagaku, Co., Ltd. (Japan). Molecular weight standards for gel filtration chromatography were from GE Healthcare Japan. All other reagents were of analytical or molecular biology grade.

### Cloning and Expression of *MalY* Gene from Genome of *L. sakei* Strain LT-13 into Cells of *E. coli* BL21 (DE3)

The *Ls-malY* gene was amplified by polymerase chain reaction (denaturing, 10 s at 98°C; annealing, 30 s at 58°C; elongation, 1 min 30 s at 68°C; 30 cycles) using *L. sakei* LT-13 chromosomal DNA as a template with the primers MalY F and MalY R (**Table 1**). Since the amplified DNA fragment contained two NdeI sites at 5′ terminus and in the coding region of *Ls-malY* gene, the fragment was digested with XhoI and partially with NdeI and then was subjected to agarose gel electrophoresis. The desired DNA fragment of approximately 1.2 kb was extracted from the gel, purified, and ligated into a pET-22b (+), yielding pE-MalY. *E. coli* BL21(DE3) cells harboring pE-MalY was cultivated in auto-induction medium (Grabski et al., 2005; Studier, 2005) containing ampicillin (100 μg/mL). After cultivation at 30°C for 24 h, cells were harvested by centrifugation at 10,000 ×*g* at 4°C for 5 min.

**Table 1:** Primers used in this study.

Primer	Sequence (5′ to 3′)
MalY F	TTCGATAGCATATG^a^ACGAAGTTTGACTTTG
MalY R	TTTCAGCTCGAG^b^TCTCTGCTGGATAGCTTC
Y123A F	TAGTCCTTGTGCGGATGCGTTTATTAATAC
Y123A R	AAACGCATCCGCACAAGGACTAAAAGTAAC
K233A F	TCTGCCAGCGCGTCATTTAATATCCCAGC
K233A R	ATATTAAATGACGCGCTGGCAGACGTTATC

*NdeI^*a*^ and XhoI^*b*^ sites are underlined*.

### Site-Directed Mutagenesis

Two plasmids for expression of *Ls*-MalY single point mutants, pE-MalY Y123A and pE-MalY K233A, were prepared from pE-MalY using a quick-change mutagenesis method with primers listed in **Table 1**. The presence of the mutation and fidelity of the mutagenesis was confirmed by sequencing. The mutated *malY* genes were expressed as described for the wild-type (WT) gene.

### Purification of *Ls*-MalY and Mutants

The harvested transformant cells were resuspended in a 20 mM potassium phosphate buffer (pH 7.4) containing 0.5 M KCl and 20 mM imidazole (Buffer A) and disrupted by ultrasonication and centrifuged to remove cell debris. The supernatant was applied to a column of Ni Sepharose^TM^ 6 Fast Flow resin (4 mL bed volume, GE Healthcare Japan) that previously equilibrated with Buffer A. After the column was washed with Buffer A, the enzyme was eluted with 20 mM potassium phosphate buffer (pH 7.4) containing 0.5 M KCl and 0.2 M imidazole. The purified enzyme was dialyzed against 20 mM potassium phosphate buffer (pH 7.5) and stored at -80°C until use.

### Gel Filtration Chromatography

The molecular weight of *Ls*-MalY was identified by size-exclusion chromatography using an ÄKTA purifier system (GE Healthcare Japan) with a Superdex 200 Increase 10/300 GL column (GE Healthcare Japan). Potassium phosphate buffer (20 mM, pH 7.5) containing 0.15 M KCl (Buffer B) was used as the isocratic mobile phase, and the flow rate was 0.75 mL/min. Thyroglobulin, apoferritin, β-amylase, bovine serum albumin (BSA), and carbonic anhydrase (150–200 μg/protein) were dissolved in Buffer B and used as a molecular weight marker.

### Enzyme Assay

The standard assay conditions for analysis of *Ls*-MalY racemase activity were as follows: reaction mixture (1 mL) containing a 50 mM potassium phosphate (pH 7.5), 50 mM substrate, 50 μM PLP and *Ls*-MalY (200 μg), incubated at 30°C for 60 min. After stopping the reaction by boiling, the supernatant was subjected to high-performance liquid chromatography (HPLC) analysis, which was performed as described previously (Gogami et al., 2011; Kato et al., 2015; Washio et al., 2016).

The β-lyase activity of *Ls*-MalY was assayed by quantifying α-keto acid using the 3-methyl-2-benzothiazolone hydrazone (MBTH) method (Soda, 1968). The standard assay conditions were as follows: after incubation at 30°C for 60 min, the reaction mixture (1 mL) consisted of a 50 mM potassium phosphate (pH 7.5), 50 mM substrate, and 50 μM PLP and *Ls*-MalY (200 μg) was mixed with 100 μL of 25% (w/v) trichloroacetic acid to stop the reaction. Sodium acetate buffer (1 M; pH 5.0; 1900 μL) and 800 μL of 0.1% (w/v) MBTH were added to the mixture, and the mixture was incubated at 50°C for 30 min. After further incubation at room temperature for 20 min, the absorbance of the mixture was measured at 316 nm.

### pH-Activity and Temperature-Activity Profiles

The effects of pH and temperature on racemase or β-lyase activity were examined using L-Ala or L-Cys as a substrate, respectively. The optimum pH for enzyme activity was determined by assaying the enzyme at 30°C at pH 4.0 to 12.0 [pH 4.0–12.0 (50 mM Britton-Robinson); pH 4.0–6.0 (50 mM acetate); pH 6.0–8.0 (50 mM potassium phosphate), pH 9.5–10.0 (50 mM borate), and pH 10.0–11.0 (50 mM carbonate)]. The optimum temperature was analyzed at pH 7.5 using 50 mM potassium phosphate from 20 to 55°C.

### Kinetic Analysis

The *Ls*-MalY enzyme reaction was performed at 40°C (racemase reaction) or 35°C (β-lyase reaction) with a reaction mixture consisting of 50 mM borate buffer (pH 10.0), 50 μM PLP, *Ls*-MalY (200 μg), and substrate. The reaction time was 10–120 min and the substrate concentrations were 0.5, 1, 2, 5, 10, 20, or 50 mM for the racemase reaction and 1, 2, 3, 4, 5, or 7.5 mM for the β-lyase reaction. Kinetic parameters for each reaction were determined using a Lineweaver-Burk plot (Lineweaver and Burk, 1934).

### Structural Modeling

A structural model of *Ls*-MalY was created with modeler software ver. 9.15 using the *E. coli* MalY (*Ec*-MalY) structure (PDB code, 1D2F) as a template.

### Spectral Analysis

Spectral changes during the β-lyase reaction of WT *Ls*-MalY with L- or D-Cys were analyzed by measuring the UV-vis absorption spectrum of the reaction mixture incubated at 30°C, which consists of 50 mM potassium phosphate (pH 7.5), *Ls*-MalY (2 mg/mL), and substrate. Dose dependence was assessed by measuring the spectrum after incubation for 30 min at a substrate concentration range of 0 to 10 mM. Reaction time-dependent changes in the spectrum of *Ls*-MalY with 10 mM substrate were monitored from 0 to 60 min.

## Results

### Identification of a Candidate Gene Related to D-Amino Acid Metabolism

**FIGURE 1:**
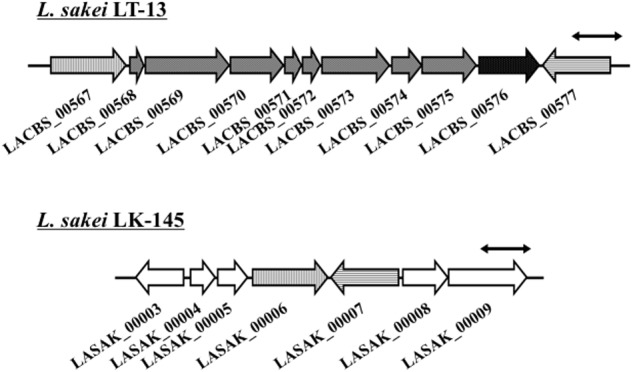
Genome map near *malY* gene of *Lactobacillus sakei* LT-13. A portion of *L. sakei* LT-13 genome near *malY* gene was compared to counter-region of *L. sakei* LK-145 genome. Solid bar with double arrowheads indicates 1 kb length. LACBS_00567: drug:H(+) antiporter; LACBS_00568: PRD domain protein; LACBS_00569: PRD domain protein; LACBS_00570: aminopeptidase; LACBS_00571: PTS system lactose/cellobiose-specific iia; LACBS_00572: PTS system lactose/cellobiose-specific iib; LACBS_00573: PTS system lactose/cellobiose-specific iic; LACBS_00574: RDD family protein; LACBS_00575: aminopeptidase; LACBS_00576: maltose regulon modulator; LACBS_00577: glutathione reductase; LASAK_00003: zinc-containing alcohol dehydrogenase; LASAK_00004: MarR family transcriptional regulator; LASAK_00005: hypothetical protein; LASAK_00006: drug:H(+) antiporter; LASAK_00007: glutathione reductase; LASAK_00008: glycine/betaine/carnitine/choline ABC transporter ATP-binding subunit; and LASAK_00009: glycine/betaine/carnitine/choline ABC transporter substrate binding protein/permease.

From the comparison of putative metabolic pathways constructed using the KEGG automatic annotation server (Moriya et al., 2007), no difference was observed in the D-amino acid metabolic pathway of *L. sakei* strains LT-13 and LK-145. However, a remarkable difference was observed in the putative Cys/Met metabolic pathway: a putative cystathionine β-lyase was identified only in the expected pathway of strain LT-13. The strain LT-13 genome contains an approximately 8-kb insertion region, including a putative cystathionine β-lyase gene *malY* (LACBS_00576), compared to strain LK-145 (**Figure 1**). Cystathionine β-lyase (EC 4.4.1.8) catalyzes a reaction that degrades L-cystathionine into L-homocysteine, ammonia and pyruvate, but no putative pathway for the biosynthesis of L-cystathionine and degradation/utilization of L-homocysteine was conserved in the strain LT-13 pathway. Overexpression of the cystathionine β-lyase gene (*malY*) from *E. coli* partially compensates the growth defect of the D-Ala-auxotrophic strain of *E. coli* (Kang et al., 2011), but the details remain unknown. Therefore, to examine the enzyme function of *Ls*-MalY, the *Ls-malY* gene was cloned and overexpressed in *E. coli*.

### Purification, Molecular Weight Analysis, and Spectral Measurement of *Ls*-MalY

**FIGURE 2:**
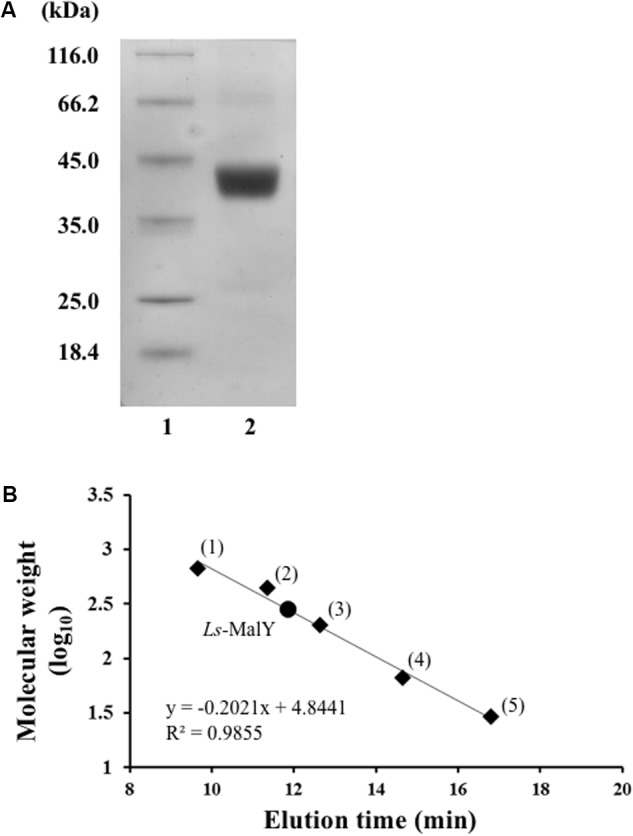
SDS-PAGE **(A)** and gel filtration chromatographic **(B)** analyses of WT *Ls*-MalY. **(A)** Lanes 1 and 2 indicate molecular weight marker and purified *Ls*-MalY (4 μg), respectively. **(B)** Thyroglobulin (1: 669,000 Da), apoferritin (2: 443,000 Da), β-amylase (3: 200,000 Da), BSA (4: 66,000 Da), and carbonic anhydrase (5: 23,000 Da) were used as molecular weight markers.

**FIGURE 3:**
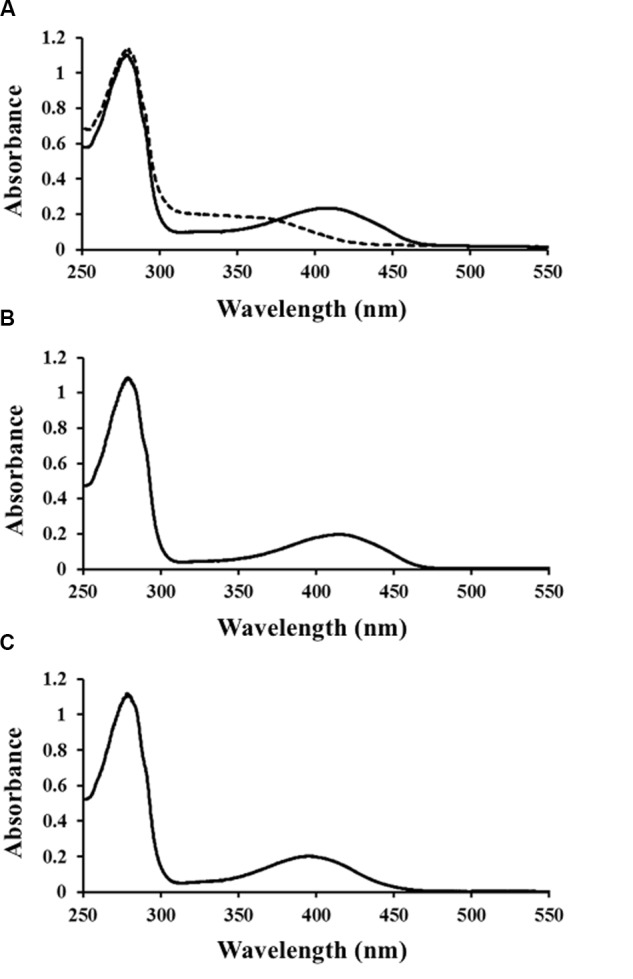
Spectral characteristics of WT **(A)**, Y123A **(B)**, and K233A **(C)** *Ls*-MalY. UV-vis spectrum of *Ls*-MalY. Solid and dashed lines indicate non-treated *Ls*-MalY and enzyme treated with 1 mM hydroxylamine in 20 mM potassium phosphate buffer (pH 7.5), respectively.

*Ls*-MalY overproduced in *E. coli* was purified to homogeneity using Ni-NTA affinity column chromatography (**Figure 2A**). From gel filtration column chromatography analysis, the molecular weight of *Ls*-MalY was estimated to be approximately 281 kDa (**Figure 2B**). The molecular weight of the *Ls*-MalY subunit with a C-terminal hexa-histidine-tag deduced from the amino acid sequence was approximately 46 kDa, suggesting that *Ls*-MalY is a homohexamer. The UV-vis absorption spectrum of purified *Ls*-MalY exhibited an absorption peak near 420 nm, and the absorption peak was abolished by treatment with hydroxylamine (**Figure 3A**), suggesting that *Ls*-MalY bound PLP.

### Reactivity and Substrate Specificity of *Ls*-MalY

**FIGURE 4:**
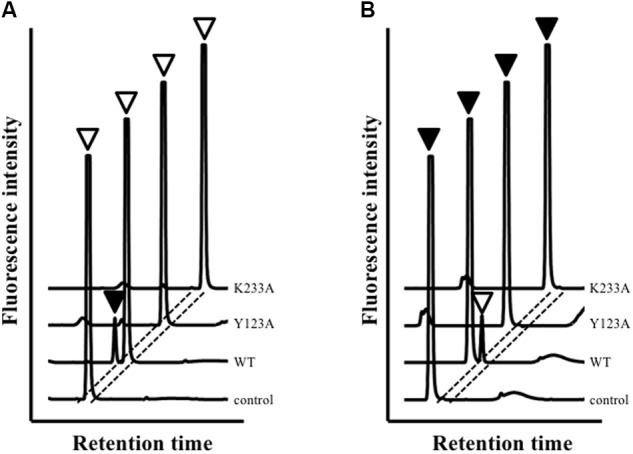
High-performance liquid chromatography (HPLC) analysis of racemase reaction toward L-Ala **(A)** and D-Ala **(B)**. White and black arrowheads indicate L-Ala and D-Ala peaks, respectively.

High-performance liquid chromatography analysis showed that *Ls*-MalY can react with L-Ala and D-Ala and catalyze the racemization reaction (**Figure 4**). The substrate specificity of *Ls*-MalY was assessed against proteinogenic amino acids for racemase activity and against Cys and Ser for β-lyase activity. *Ls*-MalY exhibited racemase activity with low substrate specificity (**Table 2**) and showed β-lyase activity toward L-Cys (**Table 3**). *Ls*-MalY also showed β-lyase activity with L-cystine and L-cystathionine (data not shown). However, the specific activity could not be calculated due to the insolubility of the substrates and products. These results indicate that *Ls*-MalY is a bifunctional amino acid racemase.

**Table 2:** Substrate specificity of racemase reaction of WT enzyme.

Substrate	Direction	
	L → D	D → L
Ala	9.3 ± 0.3	9.3 ± 0.5


Ser	1.8 ± 0.1	1.8 ± 0.1


Thr	0.059 ± 0.002	nd


Allo-Thr	0.024 ± 0.001	nd


Met	1.9 ± 0.1	1.5 ± 0.1


Glu	0.055 ± 0.007	0.097 ± 0.064


Trp	0.22 ± 0.01	0.10 ± 0.03


Tyr	Trace	Trace


Val	2.5 ± 0.1	2.4 ± 0.1


Leu	0.13 ± 0.01	0.096 ± 0.005


Asn	0.71 ± 0.09	0.44 ± 0.01


Gln	0.57 ± 0.01	0.57 ± 0.01


Lys	0.92 ± 0.06	1.0 ± 0.1


Arg	7.6 ± 0.1	5.8 ± 0.1


His	1.4 ± 0.1	1.4 ± 0.1

*The given numbers are the average of triplicate measurements and shown as specific activity (nmol/min/mg). Enzyme assay was performed under standard assay conditions. Other amino acids (Asp, Pro, Phe, Ile, and allo-Ile) were inert as substrate. nd, not detected*.

**Table 3:** Substrate specificity of β-lyase reaction.

Substrate	WT	Y123A	K233A
L-Cys	95 ± 1	0.32 ± 0.11	nd


D-Cys	nd	nd	nd


L-Ser	nd	0.42 ± 0.11	nd


D-Ser	nd	nd	6.4 ± 0.1

*The numbers are the average of triplicate measurements and shown as specific activity (nmol/min/mg). Enzyme assays were performed under the standard assay condition. nd, not detected*.

### Effects of Temperature and pH on *Ls*-MalY Activity

**FIGURE 5:**
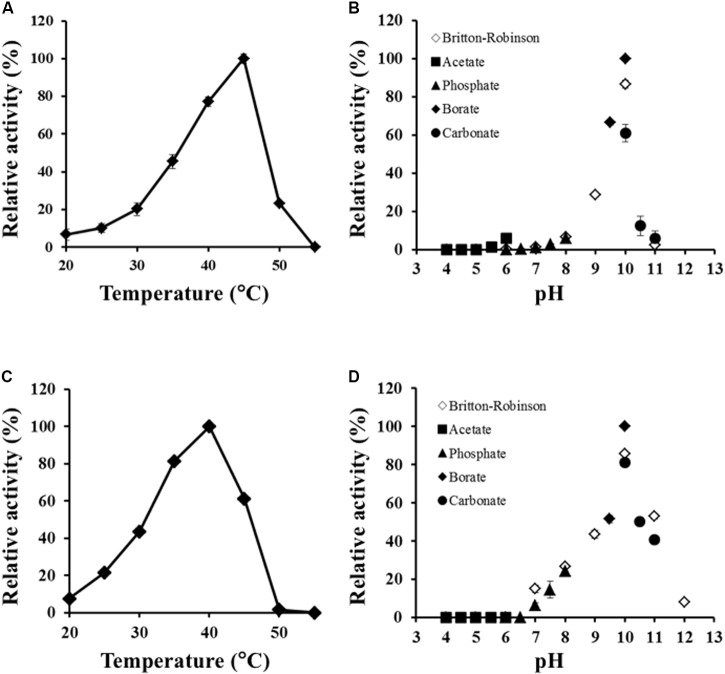
Thermal and pH profiles of racemase and β-lyase reactions. Effect of temperature on racemase **(A)** and β-lyase **(C)** reactions at pH 7.5 (50 mM potassium phosphate) and pH dependence of racemase **(B)** and β-lyase **(D)** reactions at 30°C were assessed from 20 to 55°C and pH from 4.0 to 12.0. The indicated values are the average of triplicate measurements.

Effects of temperature and pH on *Ls*-MalY activity were examined, ranging from 20 to 55°C and from pH 4.0 to 12.0. The optimal temperatures of the racemase and β-lyase reactions were 45 and 40°C, respectively (**Figures 5A,C**). The pH value optimum was the same for both reactions namely, pH 10.0 (**Figures 5B,D**).

### Kinetic Analysis of *Ls*-MalY

The kinetic parameters of *Ls*-MalY are listed in **Table 4**. From these parameters, the *K*_eq_ value of the racemization reaction between L-Ala and D-Ala was calculated as 1.12, indicating that the enzyme is a racemase (Briggs and Haldane, 1925). The *k*_cat_/*K*_m_ values for the racemase reaction and β-lyase reaction showed the same order of magnitude. *Ls*-MalY appears to act as both amino acid racemase and β-lyase, at least under the enzyme preferred conditions.

**Table 4:** Kinetic parameters of racemase and β-lyase reactions.

Reaction	Substrate	*k*_cat_ (s^-1^)	*K*_m_ (mM)	*k*_cat_*/K*_m_ (s^-1^ mM^-1^)
Racemase	L-Ala	2.31 ± 0.51 × 10^3^	169 ± 27	13.7
Racemase	D-Ala	1.83 ± 0.15 × 10^3^	150 ± 24	12.2
β-Lyase	L-Cys	9.30 ± 2.05 × 10^2^	11.5 ± 1.1	80.9

*Enzyme assay was performed at pH 10.0 and 40°C (racemase reaction) or 35°C (β-lyase reaction). Kinetic parameters were calculated with triplicate measurements*.

### Structural Modeling and Identification of *Ls*-MalY Catalytic Residues

**FIGURE 6:**
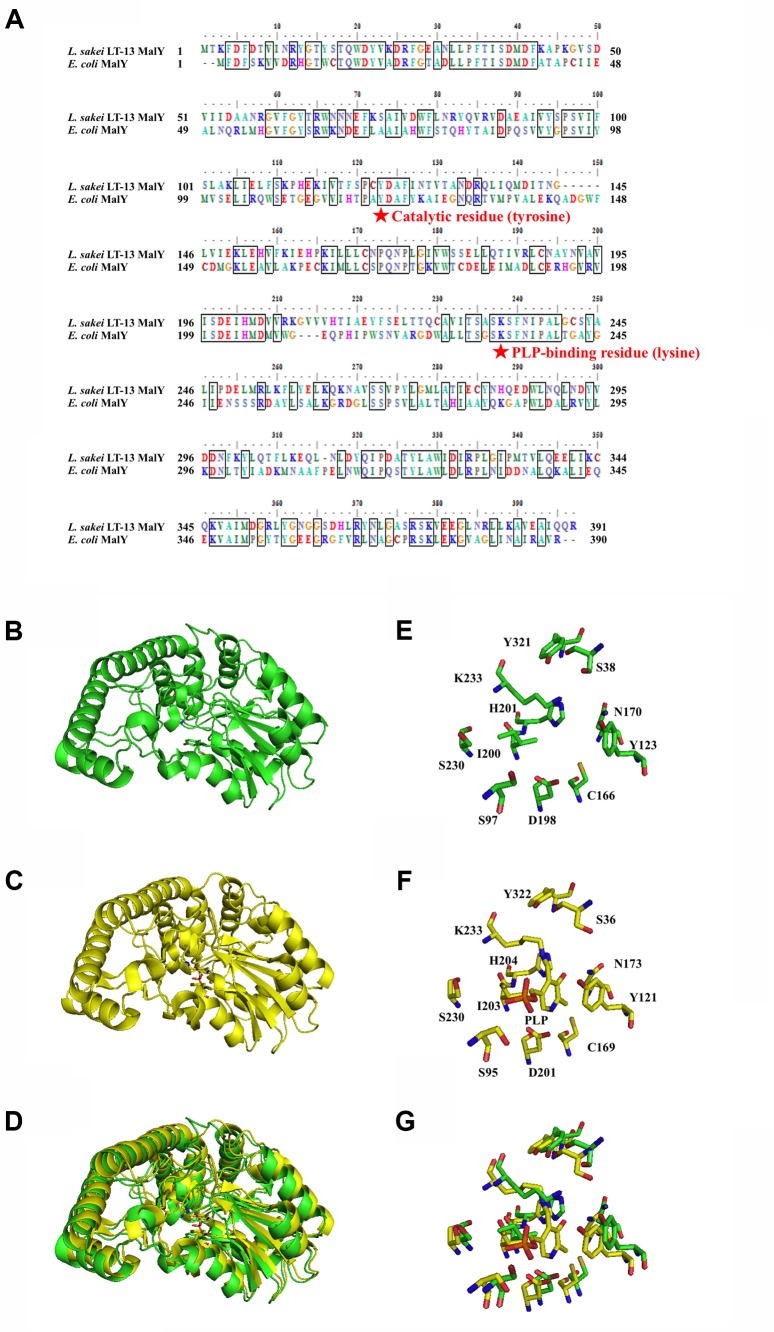
Amino-acid alignment and structural modeling. **(A)** Amino-acid alignment of *Ls*-MalY and *Ec*-MalY. **(B)** Structural model of *Ls*-MalY. **(C)** *Ec*-MalY structure (PDB code, 1D2F). **(D)** Merged view of *Ls*-MalY and *Ec*-MalY. Catalytic site of *Ls*-MalY **(E)** and *Ec*-MalY **(F)**, and its merged view **(G)**. These figures were created by GENETYX software ver. 12 **(A)** or PyMOL software ver. 0.99 **(B–G)**.

The amino-acid sequence of *Ls*-MalY showed high homology with *Ec*-MalY (identity, 42%; similarity, 60%) (**Figure 6A**). Comparison of the structural model of the *Ls*-MalY subunit created in this study and *Ec*-MalY subunit structure (Clausen et al., 2000) suggests that the overall subunit structure and amino acid configuration near the putative catalytic site of *Ls*-MalY was quite similar to *Ec*-MalY (**Figures 6B–G**). To identify catalytic residues for the racemase and β-lyase reactions, two single-point mutants (Y123A and K233A) were prepared. K233 of *Ls*-MalY is a counterpart of PLP-bound K233 of *Ec*-MalY and Y123 of *Ls*-MalY (Y121 of *Ec*-MalY) is located across the pyridine ring of PLP from K233 (**Figures 6E–G**). A spectral characteristic of the Y123A mutant (**Figure 3B**) was quite similar to the WT (**Figure 3A**), but an absorption peak derived from PLP showed a slight blueshift in the UV-vis absorption spectrum of K233A mutant (**Figure 3C**). These spectral features suggest that the Y123A mutant bound PLP and the K233A mutant contained free PLP. Both mutants lost all racemase activity (**Figure 4**), suggesting that Y123 and K233 are critical residues for the racemase reaction. Y123A mutation also caused a drastic decrease in β-lyase activity, and the K233A mutant lost β-lyase activity toward L-Cys (**Table 3**). K233 is also critical for the β-lyase reaction with L-Cys, and Y123 appears to be an important residue for the reaction. The K233A mutant and Y123A mutant showed β-lyase activity against D-Ser and L-Ser, respectively. These results suggest that K233 and Y123 are responsible for the C^α^ proton abstraction of L-enantiomers and D-enantiomers, respectively.

### Spectral Analysis of *Ls*-MalY β-Lyase Reaction

**FIGURE 7:**
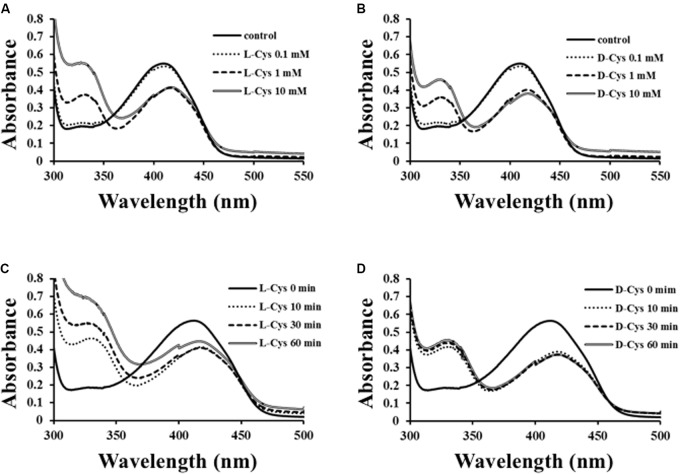
Spectral analysis of β-lyase reaction. Dose-dependent **(A,B)** and reaction time-dependent **(C,D)** changes in UV-vis spectrum of WT *Ls*-MalY during β-lyase reaction with L- or D-Cys were assessed from 0 to 10 mM and from 0 to 60 min, respectively.

To solve the reaction mechanism for *Ls*-MalY, alterations in the UV-vis absorption spectrum at 300–500 nm for WT *Ls*-MalY during the β-lyase reaction with L- or D-Cys were examined. An absorption peak at approximately 420 nm decreased dependent on the dose of substrate and a peak at approximately 330 nm, which might be derived from a reaction intermediate that simultaneously increased when reacted with L-Cys (**Figure 7A**) or D-Cys (**Figure 7B**). When reacted with L-Cys (**Figure 7C**), the spectrum was gradually altered dependent on reaction time, while the spectrum of *Ls*-MalY reacted with D-Cys was fixed after partial changes (**Figure 7D**). These results raise the possibility that L-Cys is turned over by *Ls*-MalY, whereas a deaD-end product may be formed when reacted with D-Cys.

## Discussion

The present study revealed that MalY protein from *L. sakei* LT-13 is a bifunctional enzyme that can catalyze the amino acid racemase reaction and β-lyase reaction. The enzyme preferred a moderate temperature for both reactions, in agreement with the *L. sakei* growth temperature (30–37°C). The preferred pH for enzyme activity was in the alkaline range, although the suitable condition for *L. sakei* growth is a weakly acidic environment (pH 6.0–6.5). These properties are often found in other PLP-dependent amino acid racemases derived from lactic acid bacteria (Kato et al., 2012; Mutaguchi et al., 2016), and *Ls*-MalY can display both activities in a weakly acidic condition, suggesting that *Ls*-MalY might act as a bifunctional enzyme in *L. sakei* growing cells.

The MalY protein whose enzyme characteristics are most studied is the protein from *E. coli.* *Ec*-MalY has been shown to possess β-lyase activity (Zdych et al., 1995). The PLP bound K233 of *Ec*-MalY is an essential residue for abstraction of the substrate C^α^ proton during the β-lyase reaction, and the Y121 of *Ec*-MalY interacting with pyridine ring of PLP contributes to stabilization of the carbanionic intermediate (Zdych et al., 1995; Clausen et al., 2000). These two residues conserved in **Ls*-MalY* (K233 and Y123) were shown to be important for both the amino acid racemase and β-lyase reactions by mutational analysis, suggesting that both reactions catalyzed by *Ls*-MalY share common residues for catalysis and that the mechanism of the *Ls*-MalY catalyzed β-lyase reaction is same as for *Ec*-MalY. From the configuration of K233 and Y123 in the *Ls*-MalY structural model and the results of the present mutational analysis, the *Ls*-MalY catalyzed racemase reaction appears to proceed through a two-base mechanism similar to that of the well-known alanine racemase (Watanabe et al., 2002). Some PLP-dependent enzymes are known to be inhibited by L-Cys and/or D-Cys through the formation of thiazolidine derivatives (Schonbeck et al., 1975; Dunlop and Neidle, 2005; Lowther et al., 2012). The spectral features of *Ls*-MalY when reacted with D-Cys are in agreement with such reports, suggesting that *Ls*-MalY forms a deaD-end product, namely, a thiazolidine adduct, and that *Ls*-MalY can catalyze β-lyase reactions against L-Cys but not racemase reactions between L-Cys and D-Cys. *Ec*-MalY has also been suggested to be involved in D-amino acid metabolism in *E. coli* (Kang et al., 2011), but enzyme activity toward D-amino acids has not been reported. To our knowledge, this is the first report that shows amino acid racemase activity for the MalY family protein.

*Ls*-MalY was identified from the *L. sakei* strain LT-13, which is a low-level producer of D-amino acids. In contrast, a D-amino acids high producer, the strain LK-145, does not possess MalY protein. *Ls*-MalY exhibited the highest racemase activity against Ala, and *L. sakei* LT-13 possesses a putative alanine racemase gene, suggesting that the low D-amino acid producer strain LT-13 has two enzymes that can catalyze interconversion between L-Ala and D-Ala. In *Salmonella typhimurium*, there are also two alanine racemases, Alr and DadB, which have different physiological roles: Alr and DadB are required for anabolic function in peptidoglycan assembly and cell growth on L-Ala, respectively (Walsh, 1989). The two *E. coli* alanine racemases, Alr and DadX are also involved in various events, including the biosynthesis of D-Ala for peptidoglycan and catabolism of D-Ala (Wild et al., 1985; Lobocka et al., 1994). *Ls*-MalY may act as DadB or DadX in *L. sakei* LT-13 cells. The MalY protein is conserved in some species of the *Lactobacillus* genera, including *Lactobacillus casei,* and the protein has been shown to possess β-lyase activity toward some sulfur-containing amino acids; however, reactivity toward D-amino acids has not been reported (Irmler et al., 2008). The relationship between MalY function and D-amino acid metabolism for the *Lactobacillus* genera is of interest.

The *Ls-malY* gene (LACBS_00576) is located in a putative 8-kb gene cluster, which is not conserved in strain LK-145. The cluster contains 9 genes (LACBS_00568 to LACBS_00576) encoding proteins expected to be involved in the phosphotransferase system (PTS), based on their primary structure. In *E. coli*, the *malY* gene is in *malXY* operon near its repressor *malI* gene, and the MalX protein is an enzyme relates to PTS (Reidl and Boos, 1991). *Ec*-MalY is regarded as a maltose regulon repressor and interacts with MalT protein (Schlegel et al., 2002), which is an essential transcriptional activator of the maltose regulon (Boos and Shuman, 1998). An endogenous ligand for *Ec*-MalY that is important for controlling MalT function remains unclear (Clausen et al., 2000), and the reactivity of *Ls*-MalY toward D-amino acids presented here raises the possibility that D-amino acids or homologous compounds may be native ligands for the MalY protein. The *Ls-malY* gene exists in the genome with PTS-related genes similar to the *E. coli* *malY* gene. However, no candidate gene encoding a homologous protein to MalT from *E. coli* is observed in the *L. sakei* LT-13 genome, and genes corresponding to the maltose regulon of *E. coli* are not fully conserved. We are currently investigating the physiological function(s) of MalY in *L. sakei* LT-13 cells.

## Author Contributions

TO planned this research and organized the entire manuscript. SK did all the practical experiment of this research.

## Conflict of Interest Statement

The authors declare that the research was conducted in the absence of any commercial or financial relationships that could be construed as a potential conflict of interest.
